# Integrated Analysis of DNA Methylation and Biochemical/Metabolic Parameter During the Long-Term Isolation Environment

**DOI:** 10.3389/fphys.2019.00917

**Published:** 2019-07-24

**Authors:** Yuan Quan, Fengji Liang, Yuexing Zhu, Ying Chen, Zi Xu, Fang Du, Ke Lv, Hailong Chen, Lina Qu, Ruifeng Xu, Hong-Yu Zhang, Jianghui Xiong, Yinghui Li

**Affiliations:** ^1^State Key Laboratory of Space Medicine Fundamentals and Application, China Astronaut Research and Training Center, Beijing, China; ^2^Lab of Epigenetics and Advanced Health Technology, SPACEnter Space Science and Technology Institute, Shenzhen, China; ^3^School of Computer Science and Technology, Shenzhen Graduate School, Harbin Institute of Technology, Shenzhen, China; ^4^Hubei Key Laboratory of Agricultural Bioinformatics, College of Informatics, Huazhong Agricultural University, Wuhan, China

**Keywords:** multiple time point detected data, long-term isolation environment, DNA methylation, biochemical/metabolic parameters, diseases

## Abstract

Numerous studies have shown that changes in the epigenome are an important cause of human biochemical or metabolic parameter changes. Biochemical/metabolic parameter disorders of the human body are usually closely related to the occurrence of disease. Therefore, constructing credible DNA methylation site-biochemical/metabolic parameter associations are key in interpreting the pathogenesis of diseases. However, there is a lack of research on systematic integration analysis of DNA methylation with biochemical/metabolic parameter and diseases. In this study, we attempted to use the four-people, multiple time point detected data from the long-term isolation experiment to conduct a correlation analysis. We used the biclustering algorithm FABIA to cluster the DNA methylation site-parameter correlation matrixes into 28 biclusters. The results of the biological function analysis for these biclusters were consistent with the biochemical/metabolic parameter change characteristics of the human body during long-term isolation, demonstrating the reliability of the biclusters identified by our method. In addition, from these biclusters, we obtained highly credible biochemical/metabolic parameter-disease associations, which is supported by several studies. Our results indicate that there is an overlap of biochemical/metabolic parameter-disease associations derived from a small sample, multiple time point data in healthy populations and the associations obtained from a large sample data in patients during disease development. These findings provide insights into understanding the role of the epigenome in biochemical/metabolic parameter change and disease development and has potential applications in biology and medicine research.

## Introduction

DNA methylation is a representative epigenetic mechanism in the mammalian genome. This process involves methyl group transfer to the C5 position of cytosine, which forms 5-methylcytosine in the genome. DNA methylation sites regulate gene expression by recruiting proteins involved in inhibition of gene transcription or by blocking the binding of transcription factors to DNA (Moore et al., 2013). Recently, most researchers found that DNA methylation is considered an important marker of permanent gene silencing or reactivation (Mazzio and Soliman, 2012).

The DNA methylation level of different genes determines the transcriptome of cells and tissues to a certain extent, further affecting the human body’s environmental adaptations and contributing to the diversity of biochemical and physiological characteristics, and hence phenotypic variations (Illingworth et al., 2008; Mazzio and Soliman, 2012; Neidhart, 2016). Biochemical/metabolic parameter disorders of the human body are usually closely related to the occurrence of diseases (Gupta et al., 2017). Therefore, in the process of biochemical/metabolic parameter disorders developing into diseases, DNA methylation usually has an important impact. Numerous studies have demonstrated the link between DNA methylation and complex diseases such as cancer, type 2 diabetes, major depression, schizophrenia, hypertension, and cardiovascular diseases (De Smet et al., 1999; Levenson, 2010; Guerrero-Bosagna et al., 2014; Leenen et al., 2016). Therefore, constructing credible DNA methylation site-biochemical/metabolic parameter and biochemical/metabolic parameter-disease associations is key in interpreting the pathogenesis of interactions between DNA methylation and diseases. However, there is a lack of research on systematic integration analysis of methylation-biochemical/metabolic parameter associations.

To investigate the changes of human biochemical/metabolic parameters in long-term isolation environment, the four-person 180-day CELSS Experiment was conducted in June 2016 (Xu et al., 2018). This experiment explored the change characteristics of physiology, psychology and behavior of volunteers during long-term isolation. The measured data included brain function, blood DNA methylation, cardiovascular function, biological rhythm, and sleep information (Xu et al., 2018). The DNA methylation and biochemical/metabolic parameter data accumulated in this experiment have the advantages of continuous tracking, multiple time point detection, and multidimensional omics. Therefore, we use these data for the integrated analysis of methylation and biochemical/metabolic parameter correlation.

In this study, using the four-person, multiple time point detected DNA methylation and biochemical/metabolic parameter data, we built biclusters for the four volunteers based on the FABIA algorithm (Hochreiter et al., 2010). Different biclusters have different biological functions. The same bicluster contains both DNA methylation sites and biochemical/metabolic parameters. Therefore, different biochemical/metabolic parameter-related methylation genes can be obtained according to the module distribution of the methylation and biochemical/metabolic parameter. Due to differences of gender (three males and one female), age (from 29 to 43 years), genetic backgrounds, and group roles, different volunteers can respond differently in the same external environment of long-term isolation, including DNA methylation, biochemical and metabolic parameters. Considering the reliability of the FABIA-derived biclusters, this study will focus on the analysis of the biological features shared among different individuals. In addition, we obtained the correlations between the biochemical/metabolic parameters and diseases by combining the disease information associated with genes.

## Materials and Methods

### Peripheral Blood Extraction and Biochemical and Epigenetic Assay

Peripheral whole blood cells were extracted from all four volunteers (three males and one female) before breakfast on the 12 sampling points (−45, −15, +2, +30, +60, +75, +90, +105, +120, +150, +175, and +210 days during the mission). After extraction, the whole blood cells were immediately treated with EDTA anticoagulation, transferred outside of the module and centrifuged (at 3,000 rpm for 15 min at 4°C) to isolate the plasma from the blood cells. The plasma parameter concentrations or counts were assayed with ELISA kits (R&B, United States) or RIA kits (R&B, United States) according to the manufacturer’s instructions.

DNA methylation profiling for the blood cells were determined using the Illumina 450k platform. All 48 samples were tested in one batch. DNA were extracted from the frozen blood samples by standard proteinase K/RNase treatment and phenol/chloroform extraction. Bisulfite modification of DNA (≥500 ng for each sample) was conducted using an EZ DNA Methylation Kit (Zymo Research) according to the manufacturer’s procedure. The Infinium Methylation 450K assay was performed according to Illumina’s standard protocol. The methylation profiling data are archived at the NCBI GEO online repository, accession GSE133118.

### Infinium HumanMethylation450 BeadChip Data Preprocessing

Raw intensity data (IDAT) files were imported into the R environment (version 3.4.1) and processed using the ChAMP and minfi packages. All analyses were performed in R using packages available from the Bioconductor project. The following probes were removed in data preprocessing:

1. Filtering probes with a detection *p*-value above 0.01 in one or more samples: 12497 probes were removed;
2. Filtering probes with a bead-count <3 in at least 5% of samples: 433 probes were removed;
3. Filtering probes with SNPs: 28046 probes were removed;
4. Filtering probes that align to multiple locations: 8393 probes were removed;
5. Filtering probes on the X and Y chromosomes: 10936 probes were removed.

Then the analysis would be proceeded with 425207 probes and 48 samples.

Since many EWAS aim to identify associations between methylation and diseases or environmental factors that have relatively small effects on the methylome (<10%), unwanted variation can be a significant problem for such studies. To minimize the technical error and its influence on the biological variation, we used samples of Day-45 and Day-15 as baseline replicates for each volunteer.

The following criteria were kept for further filtering of the most biological meaningful probes:

1. For each subject, probes with sd_*personal*_(β) (standard-deviation of β-value across sampling points) > error(β) between two pairs of replicates (Day-45 and Day-15) were kept for further analysis, as for the methylation variations of probes across the experiment were more biological meaningful than technique errors. For each subject, the summary of sd_*personal*_(β) was shown as in Table 1, and the summary of err_*personal*_(β) was shown as in Table 2. 192616 Probes fitting this criterion for all four subjects were kept for further analysis.
2. For each subject, sd_*personal*_(β) > 0.03. Using his criterion, we filtered the most variated methylation probes. 31821 probes were kept.
3. The intersect probes fitting the above 2 criteria were kept for further analysis.

**TABLE 1:** The summary of sd_*personal*_(β).

	**sd_*S01*_**	**sd_*S02*_**	**sd_*S03*_**	**sd_*S04*_**
Min.	0.0005088	0.0003659	0.0005051	0.0005218
1st Qu.	0.0064724	0.0064956	0.0066463	0.0066737
Median.	0.0128403	0.0131087	0.0128721	0.0128599
Mean.	0.0161165	0.0160469	0.0157420	0.0157409
3rd Qu.	0.0213100	0.0217733	0.0210371	0.0211823
Max.	0.3461165	0.3629779	0.3497298	0.2542050

**TABLE 2:** The summary of err_*personal*_(β).

	**err_*S01*_**	**err_*S02*_**	**err_*S03*_**	**err_*S04*_**
Min.	0.000000	0.000000	0.000000	0.000000
1st Qu.	0.001800	0.001775	0.002006	0.002214
Median.	0.004839	0.004745	0.005488	0.006678
Mean.	0.008024	0.007923	0.009936	0.012305
3rd Qu.	0.010925	0.010556	0.012715	0.016583
Max.	0.392298	0.384768	0.450809	0.359334

**FIGURE 1:**
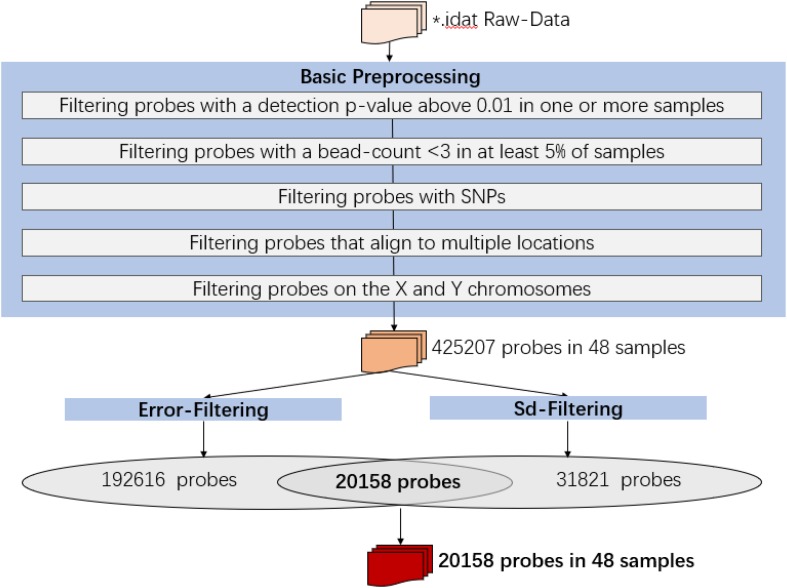
Infinium HumanMethylation450 BeadChip data preprocessing.

Finally, the data set for downstream analysis comprised 20,158 probes and 48 samples (Figure 1).

### Metabolic Assay and Biochemical Assays

During the experiment, the morning urine of Day-15, Day-30, Day-45, Day-60, Day-75, Day-90, Day-105, Day-120, Day-135, Day-150, Day-165, and Day-175 for the volunteers were collected and UHPLC-Q/Exactive high resolution mass spectrometry was used to detect molecule metabolites in urine (Dong et al., 2018). In addition, details on various biochemical assays will be described in other papers.

### Correlation Matrix Construction for DNA Methylation Sites and Biochemical/Metabolic Parameters

Using the preprocessing data of 20,158 DNA methylation sites and 223 biochemical/metabolic parameters, we used the Spearman rank correlation to calculate the correlation coefficients between each DNA methylation site and each biochemical/metabolic parameter for the four volunteers, respectively. To ensure the consistency of the DNA methylation data with the biochemical/metabolic parameter data, this study only retained the data obtained from seven time points (Day-2, Day-30, Day-60, Day-90, Day-120, Day-150, Day-175) for Spearman rank correlation calculation. As a result, we obtained four 20,158 × 223 correlation matrixes of DNA methylation sites and biochemical/metabolic parameters (Tables S1–S4). The Spearman correlation coefficients were calculated by the R script.

### Biclustering Analysis Based on FABIA

The FABIA algorithm was used to perform biclustering analysis for the above four DNA methylation site-biochemical/metabolic parameter correlation matrixes (Hochreiter et al., 2010). According to your suggestions, we have added the detailed rationales of FABIA in our revised manuscript (line139∼line148): FABIA is a multiplicative model-based biclustering approach that assumes realistic non-Gaussian signal distributions with heavy tails (Hochreiter et al., 2010). It is based on multiple model selection techniques, such as variational methods and Bayesian framework. Analysis of several real biological data proved that FABIA can determine the information content of each bicluster to separate spurious biclusters from true biclusters effectively (Hochreiter et al., 2010). This biclustering approach models the data matrix *X* as the sum of *p* biclusters plus additive noise γ, where each bicluster is the outer product of two sparse vectors: a row vector λ and a column vector *z*. The model for the matrix *X* is as follows:

$$X=\sum_{i=1}^{p}\lambda_{i}\,z_{i}^{T}+\gamma$$

Where λ*_*i*_* is the column vector of the i-th class of genes, *z_*i*_^*T*^* is the column vector of the i-th class of samples, and γ is the additive noise, respectively.

Then, to make the matrixes conform to the input criteria of the FABIA algorithm, we used the scale function in R to normalize the matrix. The processed matrixes were conformed to the standard normal distribution, i.e., the data mean is 0 and the standard deviation is 1. In our study, FABIA 2.2.2 software was used to search K biclusters of the four 20158 × 223 matrixes. K (the number of biclusters) was set to 50. The sparseness factor was set to 0.1 and the number of iterations was set to 15000. The FABIA algorithm was implemented in the “fabia” package of R script.

### Disease-Associated Gene Collection and Standardization

The disease-associated genes were obtained from eight databases, including GAD^1^, OMIM^2^, Clinvar^3^, Orphanet^4^, GWASdb^5^, NHGRI GWAS Catalog^6^, RegulomeDB^7^ and partial data from The HGMD^8^ that appeared in Wang et al.’s work (Becker et al., 2004; Hamosh et al., 2005; Boyle et al., 2012; Wang et al., 2012; Landrum et al., 2013; Welter et al., 2013; Li et al., 2015). To facilitate the subsequent analysis, the natural language processing tool MetaMap was used to convert disease terms associated with genes to UMLS concepts, and the MeSH thesaurus was selected as the UMLS vocabulary source. In addition, to prevent fragmented descriptions of diseases, we used the disease classes provided by Pharmaprojects (similarity threshold: 0.75) to standardize the diseases (Aronson, 2001). As a result, we obtained 19,233 disease genes, involving 703 types of diseases.

### Permutation Test

To assess whether the identified DNA methylation gene-biochemical/metabolic parameter associations were random results, a permutation test was performed. One thousand samples were generated by random shuffling of common DNA methylation gene-biochemical/metabolic parameter associations derived from the FABIA biclusters of the four volunteers. Then, we calculated the frequency of STITCH^9^ - validated ratios derived from the 1,000 random combinations of DNA methylation genes and biochemical/metabolic parameters (Szklarczyk et al., 2016). The frequency distributions were compared with the actual ratio of common DNA methylation gene-biochemical/metabolic parameter associations derived from the FABIA biclusters of the four volunteers.

### Bicluster Function Enrichment

The biological function KEGG pathways were enriched for each bicluster using records in the DAVID^10^ (Huang da et al., 2009). The corresponding catalogs and diseases of the KEGG pathways were downloaded from the KEGG PATHWAY Database^11^ (Qiu, 2013).

## Results and Discussion

### Biclustering Analysis for DNA Methylation and Biochemical/Metabolic Parameter

**FIGURE 2:**
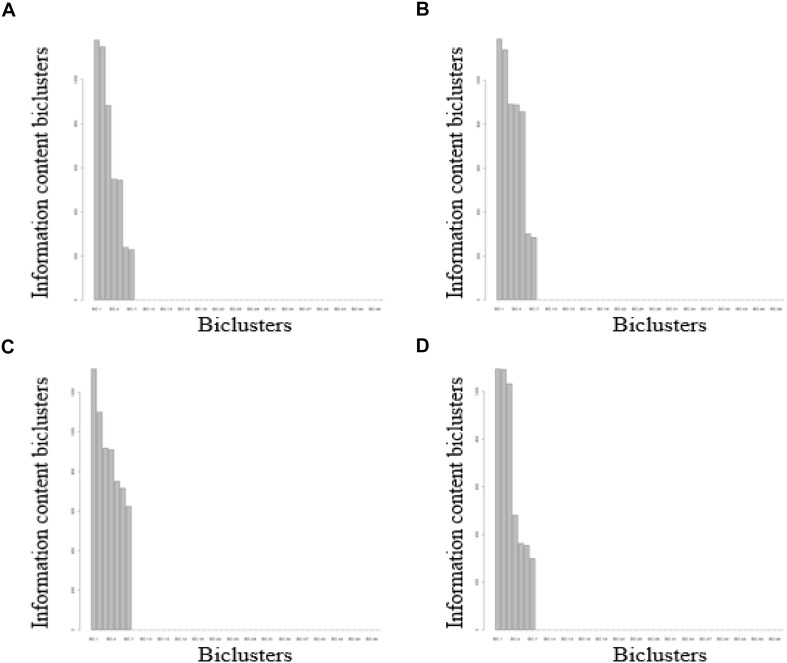
FABIA results for DNA methylation-biochemical/metabolic parameter correlation matrixes. **(A)** For volunteer A, **(B)** for volunteer B, **(C)** for volunteer C, **(D)** for volunteer D.

For the FABIA-derived biclusters of the four volunteers, when the number of biclusters (K) ≥ 8, the superfluous bicluster information content was close to zero, indicating that the biclusters contained all the information of the matrix (Figure 2) (see Materials and Methods). The biclustering process are tested on a computer with 3.60 GHz quad-core Intel Core CPUs and 8 GB main memory, which running time is about 312 h. Bicluster 1 involves the richest information and bicluster 7 involves the poorest (Figure 2). In the clustering results obtained by the FABIA method, one DNA methylation probe or one biochemical/metabolic parameter can participate in multiple biclusters.

**FIGURE 3:**
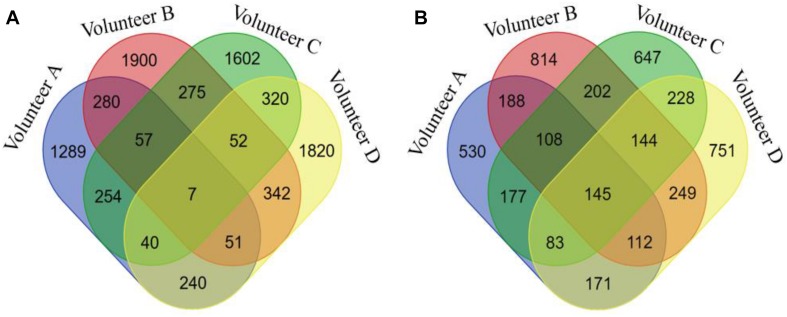
The Venn diagram of DNA methylation probes or genes contained in seven biclusters of different volunteers. **(A)** for DNA methylation probes, **(B)** for DNA methylation genes.

The seven biclusters of volunteer A contain 2,218 DNA methylation probes that can be mapped to 1,514 genes based on the annotation of the 450k chip, volunteer B consisted of 2,964 DNA methylation probes that can be mapped to 1,962 genes, volunteer C contain 2,607 DNA methylation probes that can be mapped to 1,734 genes, and volunteer D consisted of 2,872 DNA methylation probes that can be mapped to 1883 genes (Table S5). The intersection distribution of the DNA methylation probes and genes contained in the biclusters of the four volunteers are shown in Figure 3. Because multiple probes can map to the same gene, the intersection numbers of DNA methylation genes contained in the biclusters of each volunteer is greater than the number of DNA methylation probes.

**FIGURE 4:**
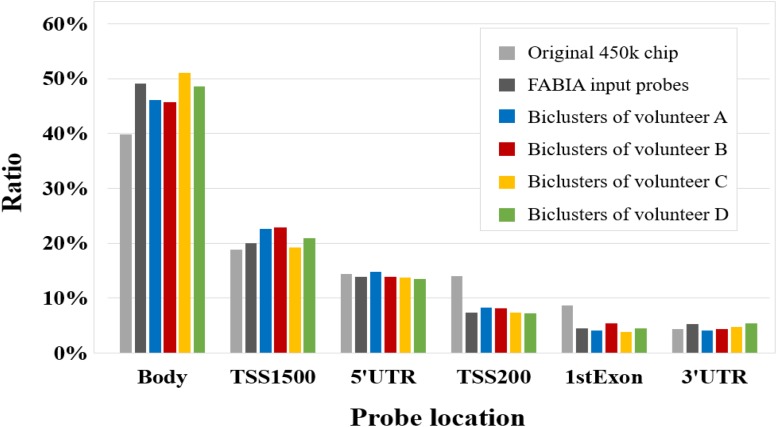
The gene positional distribution of DNA methylation probes for the original 450k chip, FABIA input probes, and FABIA-derived biclusters of the four volunteers.

In addition, we compared the corresponding gene location distribution of the DNA methylation probes for the original 450k chip, FABIA input probes, and FABIA-derived biclusters of the four volunteers. As showed in Figure 4, the ratio of the FABIA input probes located in the corresponding gene body regions was significantly higher than that of the 450k chip, and the probes contained in the FABIA-derived biclusters retained this property.

**FIGURE 5:**
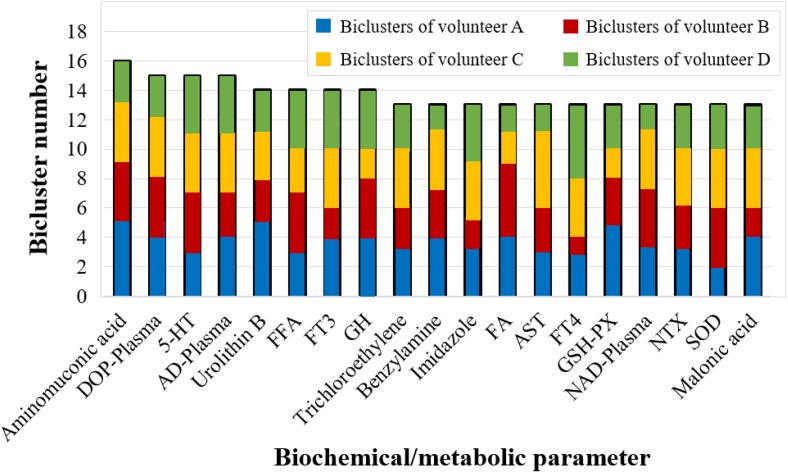
The biochemical/metabolic parameters covering more than 13 FABIA-derived biclusters (bicluster number ≥ 13).

Furthermore, we counted the number of covered biclusters for the 223 biochemical/metabolic parameters. The biochemical/metabolic parameters covering the largest number of FABIA-derived biclusters are shown in Figure 5: aminomuconic acid involved 16 biclusters, DOP-Plasma, 5-HT, AD-Plasma covered 15 biclusters, and Urolithin B, FFA, FT3, GH covered 14 biclusters (Table S5). Interestingly, these results showed that the biochemical/metabolic parameters related to mood state (DOP-Plasma, 5-HT, AD-Plasma) and glucose metabolism (aminomuconic acid, FT3 and FFA, GH) involved the largest number of biclusters, suggesting that these two parameter types have a significant change trend in 4-person 180-day data.

### KEGG Pathway and Disease Enrichment Analysis for FABIA-Derived Biclusters

**FIGURE 6:**
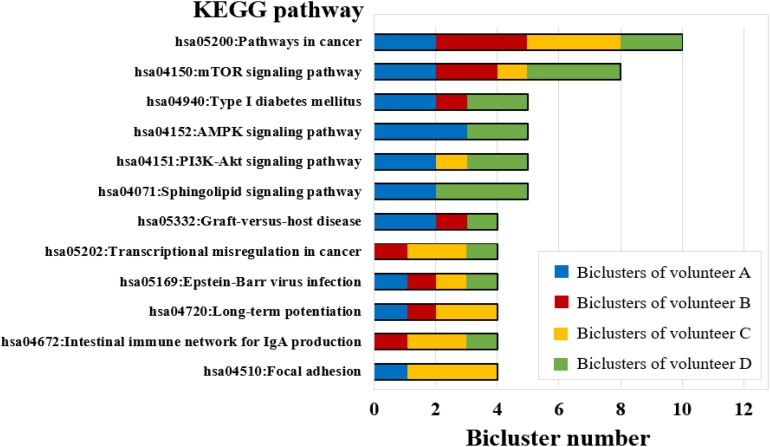
KEGG pathway enrichment analysis for FABIA-derived biclusters of the four volunteers (bicluster number ≥ 4).

The biological functions were enriched for each bicluster using the KEGG pathway records in DAVID (see Materials and Methods) (Huang da et al., 2009). The results have shown that all biclusters were enriched in 111 KEGG pathways (*P*-value ≤ 0.05) (Table S6), of which the biclusters of volunteer A involved 44 significant KEGG pathways, volunteer B and volunteer C were enriched with 41 significant KEGG pathways, and volunteer D was enriched with 49 significant KEGG pathways (Table S6). Among them, some KEGG pathways can be enriched by multiple biclusters. For instance, the pathways in cancer were involved in 10 biclusters, mTOR signaling pathway covered 8 biclusters, Type I diabetes mellitus, AMPK signaling pathway, PI3K-Akt signaling pathway, and Sphingolipid signaling pathway were involved in 5 biclusters (Figure 6). It is worth noting that PI3K-Akt signaling pathway is one of the widely studied biological pathways. Phosphatidylinositol 3-kinases (PI3Ks) is a major downstream molecule of tyrosine kinases and G-protein coupled receptors, and it can activate Akt, glycogen synthase kinase-3 (GSK-3) through catalyzing the production of a second messenger 3,4,5-triphosphate phosphatidylinositol (PIP3), that transmit signals of various growth factors and cytokines into cells (Zhang L. et al., 2018). Thus, PI3K-Akt signaling pathway plays an important regulatory role in various biological processes such as cell proliferation, differentiation, apoptosis and glucose transport (Yu and Cui, 2016). Previous studies have shown that PI3K-Akt signaling pathway is associated with the occurrence of bone diseases such as short limb skeletal dysplasia and achondrogenesis type II (Qiu, 2013). This result implies that the long-term isolation environment is unfavorable to human bones, which is consistent with common sense.

Further, a query of the KEGG PATHWAY Database showed that several above pathways are associated with the same disease type. For example, the focal adhesion pathway is related to growth delay due to insulin-like growth factor I resistance, the AMPK signaling pathway is related to leptin receptor deficiency, the type I diabetes mellitus pathway is related to type 1 diabetes mellitus, and all of these diseases can be classified as endocrine and metabolic diseases (Qiu, 2013). In addition, the focal adhesion pathway is related to Limb-girdle muscular dystrophy, which is belonged to musculoskeletal diseases (Qiu, 2013). The above results are consistent with the effects of long-term isolation on the human body in the 4-person 180-day experiment, including disturbances in the endocrine, metabolism, and musculoskeletal systems.

**FIGURE 7:**
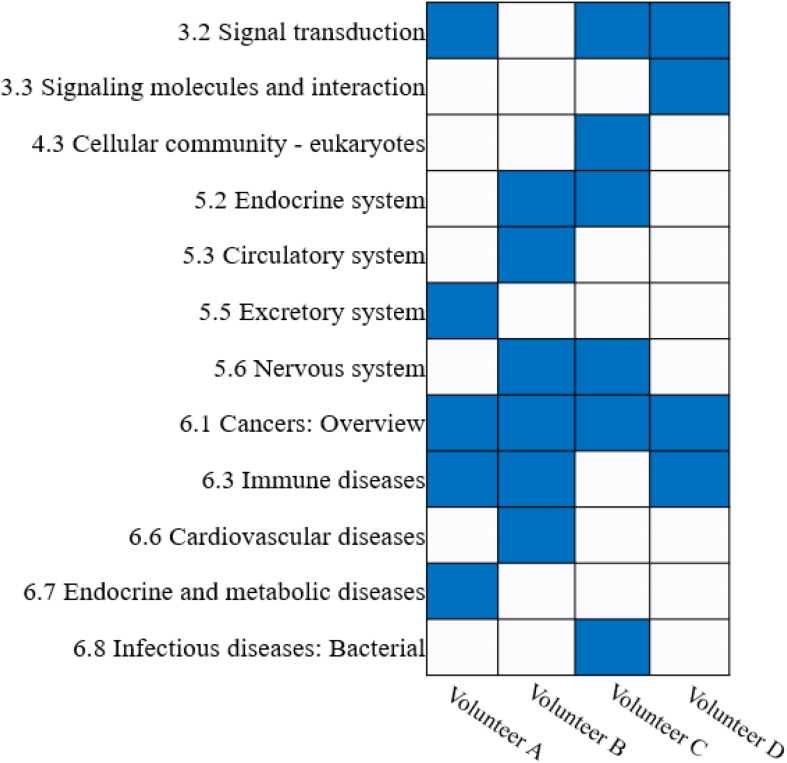
Enrichment analysis of KEGG pathway catalogs for FABIA-derived biclusters of the four volunteers.

Furthermore, we annotated the KEGG pathways with their corresponding catalogs using the KEGG PATHWAY Database (Qiu, 2013). The results show that the KEGG pathways obtained from different volunteers can be enriched in different catalogs (Hypergeometric test, *P*-value ≤ 0.05). However, there are several common catalogs that are simultaneously enriched in multiple volunteer’s biclusters, such as the cancer catalog that was enriched in all volunteers, and the immune diseases and signal transduction catalogs were enriched in three of four volunteers (Figure 7).

Next, by combining the data of 19,233 genes and their associated disease annotations from eight disease gene databases (GAD, OMIM, Clinvar, Orphanet, GWASdb, NHGRI GWAS Catalog, RegulomeDB, and HGMD) (see Materials and Methods) (Becker et al., 2004; Hamosh et al., 2005; Boyle et al., 2012; Wang et al., 2012; Landrum et al., 2013; Welter et al., 2013; Li et al., 2015), we performed a disease enrichment analysis of the FABIA-derived biclusters of the four volunteers (Hypergeometric test, *P*-value ≤ 0.05). As shown in Table 3, 13 diseases can be enriched by more than 20 biclusters. Based on the classification of diseases in the NCBI MeSH database^12^, the above 13 diseases are involved in four disease catalogs (Table 3). Among them, the number of cardiovascular diseases and nervous system diseases is the highest. Disease cataplexy, narcolepsy, restless legs syndrome, sleep disorders, sleep initiation, and maintenance disorders are nervous system diseases. Notably, four (cataplexy, narcolepsy, sleep disorders, sleep initiation, and maintenance disorders) of these diseases are related to sleep disorders. Telangiectasis, vascular diseases, ataxia telangiectasia, and unstable angina are classified as cardiovascular diseases.

**TABLE 3:** Enriched diseases of the FABIA-derived biclusters (covering ≥ 20 biclusters).

**Disease catalog^1^**	**Disease**	**Bicluster number**
Cardiovascular diseases	Telangiectasis	24
	Vascular diseases	24
	Ataxia telangiectasia	21
	Angina, unstable	20
Chemical induced disorders	Alcoholism	28
	Substance-related disorders	28
	Tobacco use disorder	28
Nervous system diseases	Cataplexy	24
	Narcolepsy	24
	Restless legs syndrome	24
	Sleep disorders	24
	Sleep initiation and maintenance disorders	23
Nutritional and Metabolic Diseases	Metabolic diseases	20

*^1^Derived from NCBI MeSH (https://www.ncbi.nlm.nih.gov/mesh).*

### Construction of DNA Methylation Site-Biochemical/Metabolic Parameter Associations

**FIGURE 8:**
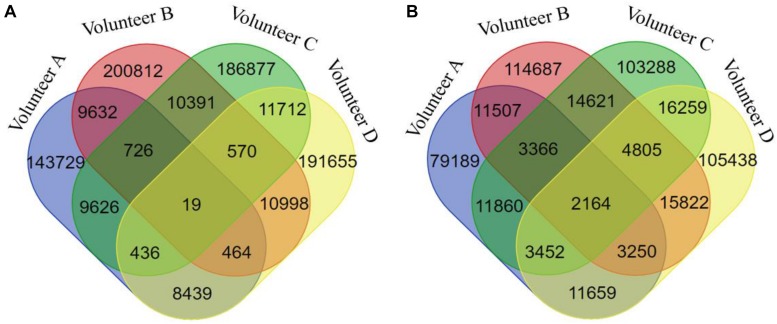
The Venn diagram of DNA methylation probe/gene-biochemical/metabolic parameter associations of different volunteers. **(A)** For DNA methylation probes, **(B)** for DNA methylation genes.

In this study, we hypothesized that a DNA methylation site/gene and a biochemical/metabolic parameter involved in the same bicluster can constitute a functional association. According to this principle, we obtained 786,086 DNA methylation site-biochemical/metabolic parameter associations based on the FABIA-derived biclusters of the four volunteers (Figure 8). To promote the reliability of these associations, we only selected the associations that occurred in at least three volunteers’ biclusters for subsequent analysis, covering 1,567 DNA methylation gene-biochemical/metabolic parameter associations, involving 153 genes and 211 biochemical/metabolic parameters (Table S7).

**FIGURE 9:**
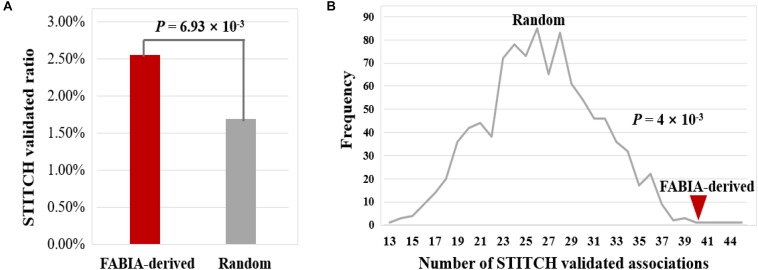
Validation of DNA methylation gene-biochemical/metabolic parameter associations using the STITCH database. **(A)** By the hypergeometric test, **(B)** by the permutation test. Lines indicate the frequency of STITCH-validated associations derived from 1,000 random combinations of DNA methylation genes and biochemical/metabolic parameters, and red triangle indicate the number of STITCH-validated associations obtained from the FABIA-derived biclusters.

Next, we verified the reliability of these 1,567 DNA methylation gene-biochemical/metabolic parameter associations by searching STITCH (Szklarczyk et al., 2016). The results show that 40 of 1567 associations were validated by the STITCH database (2.55%) (Figure 9A and Table S7). For 32283 associations of 153 genes randomly combined with 211 biochemical/metabolic parameters, this ratio is only 1.69% (547 of 32283 associations), which is significantly lower than that of the FABIA-derived DNA methylation gene-biochemical/metabolic parameter associations (*P*-value = 6.93 × 10^−3^, hypergeometric test) (Figure 9A). In addition, we performed a 1000 permutation test to verify the reliability of the FABIA-derived DNA methylation gene-biochemical/metabolic parameter associations (*P*-value = 4 × 10^−3^, permutation test) (Figure 9B) (see Materials and Methods). The results show the reliability of associations obtained in this study and deserve further study.

### Construction of Biochemical/Metabolic Parameter-Disease Associations

Based on the above DNA methylation gene-biochemical/metabolic parameter associations, and information obtained from eight disease gene databases (see Materials and Methods) (Becker et al., 2004; Hamosh et al., 2005; Boyle et al., 2012; Wang et al., 2012; Landrum et al., 2013; Welter et al., 2013; Li et al., 2015), we can enrich the diseases associated with different biochemical/metabolic parameters by the hypergeometric test. Eight thousand hundred and eight biochemical/metabolic parameter-disease associations were significantly enriched (hypergeometric test, *P*-value ≤ 0.05), involving 203 biochemical/metabolic parameters and 626 diseases (Table S8). Some of these associations are supported by the published literature. As shown in Table 4, 8 of the top 20 significant biochemical/metabolic parameter-disease associations were verified by previous studies (Rasmuson et al., 2001; Lei et al., 2011; Sunose et al., 2011; Karki et al., 2015; Baldissarelli et al., 2016; Kim et al., 2017; Zhang Y. et al., 2018).

**TABLE 4:** The most significant biochemical/metabolic parameter-disease associations (top 20).

**Biochemical/metabolic parameter**	**Disease**	***P*-values**	**Evidence**
Formic acid	Atrial fibrillation	1.56e-06	
Methylbutyrylglycine	Anemia, aplastic	1.71e-06	
Formic acid	Arrhythmias, cardiac	5.15e-06	
VA (vitamin a)	Sarcopenia	1.01e-05	Kim et al., 2017
Cortisol	Carcinoma, renal cell	1.58e-05	Rasmuson et al., 2001
Methylbutyrylglycine	Myelodysplastic syndromes	1.75e-05	
Methylbutyrylglycine	Polycythemia vera	1.76e-05	
VA (vitamin a)	Muscle spasticity	2.09e-05	
FFA (free fatty acid)	Pain	2.67e-05	Karki et al., 2015
TG (triglyceride)	Atrial fibrillation	3.49e-05	Zhang Y. et al., 2018
VEGF (vascular endothelial growth factor)	Malabsorption syndromes	4.60e-05	Lei et al., 2011
Methylnicotinamide	Memory disorders	4.80e-05	
Acetylaminooctanoic acid	Impulse control disorders	4.95e-05	
Quercetin	Thrombocytopenia	5.10e-05	Baldissarelli et al., 2016
Methylbutyrylglycine	Hematologic neoplasms	5.17e-05	
Aspartylaspartic acid	Carcinoma, neuroendocrine	6.47e-05	
GGT (gamma-glutamyltransferase)	Carcinoma, neuroendocrine	6.47e-05	Sunose et al., 2011
Acetylaminooctanoic acid	Adrenoleukodystrophy	6.98e-05	
TG (triglyceride)	Arrhythmias, cardiac	7.23e-05	Zhang Y. et al., 2018
Methylbutyrylglycine	Anemia	7.70e-05	

To further verify the reliability of the above biochemical/metabolic parameter-disease associations, this study compared the co-occurrence publication numbers of FABIA-derived associations with the random associations in the biomedical literature using NCBI Medline abstracts by text mining with The Entrez Programming Utilities (E-utilities)^13^. When a biochemical/metabolic parameter with a disease appeared in the abstract of the same publication, a co-occurrence record was retrieved. The results show that the co-occurrence numbers of 48.51% (3,933 of 8,108) associations were not zero (Table S8). The co-occurrence number of the FABIA-derived associations was significantly higher than the random associations (*P*-value = 2.20 × 10^−16^, Wilcoxon test).

The above results preliminarily proved the credibility of the biochemical/metabolic parameter-disease associations identified by the FABIA-derived biclusters. Notably, there is a certain overlap between the biochemical/metabolic parameter-disease associations obtained from healthy populations during long-term isolation and the associations obtained from patients during disease development. Therefore, the remaining associations that have not been researched in published literature are worthy of further study.

## Conclusion

In this paper, we constructed 28 biclusters using the FABIA algorithm based on the 180-day CELSS experiment-derived DNA methylation and biochemical/metabolic parameter data of four volunteers at 7 time points. The results show that the biochemical/metabolic parameters related to mood state (DOP-Plasma, 5-HT, AD-Plasma) and glucose metabolism (aminomuconic acid, FT3 and FFA, GH) involved the largest number of biclusters, suggesting that these two parameter types have a significant change trend in the long-term isolation environment. In addition, analysis of the biological functions of the genes covering each biclusters showed that these genes are significantly enriched in endocrine and metabolic diseases-related and musculoskeletal diseases-related KEGG pathways. The disease enrichment analysis revealed that the above biclusters are significantly associated with cardiovascular diseases and nervous system diseases, particularly sleep disorders. The above results for the FABIA-derived biclusters were consistent with the parameter change characteristics of the human body during the long-term isolation environment, demonstrating the reliability of the biclusters identified by our method.

Next, we obtained 1,567 DNA common methylation gene-biochemical/metabolic parameter associations from the four volunteers’ 28 biclusters, and their STITCH-validated ration is higher than that of random combinations. This indicates the value of the bicluster-derived methylation gene-biochemical/metabolic parameter associations. By combining the data of disease-related genes, we identified 8,108 biochemical/metabolic parameter-disease associations. The cooccurrence numbers in the literature based on text mining show that most of the biochemical/metabolic parameter-disease associations were supported by the existing literature. This result preliminarily proved the credibility of the biochemical/metabolic parameter-disease associations identified by our methods. Therefore, the remaining associations that have not been published in research are worthy of further study.

In this experiment, due to the ethical requirements, four volunteers maintained a healthy state and did not show any diseases or symptoms. However, the data showed that their DNA methylation level, biochemical and metabolic parameters all occurred some disturbances during the long-term isolation. Considering the reliability of these disturbances, our study was only focused on the disturbance-derived disease associations shared among different individuals. The results indicated that there is a significant overlap between the biochemical/metabolic parameter-disease associations obtained from healthy populations during long-term isolation and the associations obtained from patients during disease development. This finding provides insights into the role of the epigenome in biochemical/metabolic parameter change and disease development, and has potential applications in biology and medical research.

In this study, we hypothesized that small sample experiments also have biological significant. Our analysis shows that the biological associations obtained based on small sample experiments have indeed been supported by previous literatures. Certainly, it is not enough to validate this hypothesis just use other literatures, and the lack of independent experimental verification is one of the limitations for this study. It is a provocative hypothesis that needs to be validated by other independent experiments in the future.

In addition, although this paper can obtain some biologically meaningful biclusters based on FABIA algorithm, it is a time-consuming biclustering process. For the four 20158 × 223 matrixes of this study, 15000 iterations are required to make the superfluous bicluster information content was close to zero, and the running time of biclustering process is about 312 h (a computer with 3.60 GHz quad-core Intel Core CPUs and 8 GB main memory). Furthermore, FABIA’s biclustering results also depend on manual adjustment of initial input parameters, including the sparseness factor and number of iterations, which cannot be fully automated clustering. Therefore, there are still other more routine and simplified biclustering algorithms worthy of further attempts by researchers. We expect more interesting results to be generated based on data of small sample experiments. And this study is only used as a heuristic paper for small sample experiments. To sum up, our results indicate the potential applications of the multiple time point detected, micro-perturbed, long-term isolation and small sample experiment-obtained data in biological and medical study.

## Ethics Statement

This study was carried out in accordance with the recommendations of the Astronaut Center of China Ethics Committee with written informed consent from all subjects. The protocol was approved by the Astronaut Center of China Ethics Committee. All subjects gave written informed consent in accordance with the Declaration of Helsinki.

## Author Contributions

JX and YL designed the experiments. JX led the epigenetic research and designed the strategy for integrated analysis of DNA methylation with metabolome. FL, KL, YZ, YC, ZX, FD, and LQ performed the sample and the data collection. YQ and FL conducted the DNA methylation data mining and bioinformatics analyses. RX and H-YZ helped in preparing the manuscript. JX and YL interpreted the results. YQ wrote the manuscript.

## Conflict of Interest Statement

The authors declare that the research was conducted in the absence of any commercial or financial relationships that could be construed as a potential conflict of interest.

## Footnotes

1 https://geneticassociationdb.nih.gov/

2 http://omim.org/

3 http://www.ncbi.nlm.nih.gov/clinvar/

4 http://www.orpha.net/consor/cgi-bin/index.php

5 http://jjwanglab.org/gwasdb

6 https://www.ebi.ac.uk/gwas/

7 http://www.regulomedb.org/

8 http://www.hgmd.cf.ac.uk/ac/index.php

9 http://stitch.embl.de/

10 https://david.ncifcrf.gov/

11 https://www.genome.jp/kegg/pathway.html

12 https://www.ncbi.nlm.nih.gov/mesh

13 www.ncbi.nlm.nih.gov/books/NBK25501/

## Abbreviations

5-HT: serotonin
AD-Plasma: plasma adrenaline
CELSS: Controlled Ecological Life Support System
DAVID: Database for Annotation Visualization and Integrated Discovery
DOP-Plasma: plasma dopamine
ELISA: enzyme-linked immunosorbent assay
FABIA: factor analysis for bicluster acquisition
FFA: free fatty acid
FT3: free triiodothyronine
GAD: Genetic Association Database
GH: growth hormone
HGMD: Human Gene Mutation Database
KEGG: Kyoto Encyclopedia of Genes and Genomes
MeSH: Medical Subject Headings
OMIM: Online Mendelian Inheritance in Man
RIA: radio-immunological assay
STITCH: Chemical-Protein Interaction Networks database
UMLS: Unified Medical Language System.
